# Diphtheria of the Conjunctiva

**Published:** 1900-02-24

**Authors:** 


### DIPHTHERIA OF THE CONJUNCTIVA.

Mr. Sydney Stephenson,[1] says that in his experience ophthalmia associated with the Klebs-Loeffler bacillus is far from uncommon in London, although it must often escape recognition unless a routine bacteriological investigation be carried out. At the North-Eastern Hospital for Children, where such an examination is made in all doubtful cases, it is estimated that 2 per cent. of all recent cases of ophthalmia are of that nature. The cases run in groups, and are more frequent when diphtheria is common among the children of the district. Children over three are seldom attacked. While, however, the diphtheria bacillus is met with in this considerable proportion of the cases of ophthalmia, the classical conjunctival diphtheria described by Graefe is practically unknown at the hospital in question, which seems also to be the experience all over the country.

[1] Lancet, Feb. 17.

